# Rare association of the ovarian adenocarcinoma with pregnancy: A case report

**DOI:** 10.1016/j.amsu.2022.103350

**Published:** 2022-02-05

**Authors:** Maryem Bouab, Elhassane Elazzouzi, Fatiha El Miski, Mohamed Jalal, Amine Lamrissi, Karima Fichtali, Said Bouhya, Imane Beliamime, Ayoub Khouaja, Mehdi Karkouri

**Affiliations:** a Obstetrics and Gynecology Department, University Hospital Center Ibn Rochd, Casablanca, Morocco; b Pathology Department, University Hospital Center Ibn Rochd, Casablanca, Morocco; c Faculty of Medicine and Pharmacy, Hassan II University, Casablanca, Morocco

**Keywords:** Adenocarcinoma, Ovary, Pregnancy, Childbirth, Chemotherapy

## Abstract

The association of ovarian malignancy with pregnancy is rare; accounting for 3–6% of ovarian masses of which malignant germ cell tumors represent the type most frequently associated with pregnancy, whereas the incidence of epithelial ovarian cancer is only 1/12,000 to 1/50,000 of pregnancies. The diagnosis and management of ovarian cancer in pregnancy remain poorly codified because of the rarity of cases and the limited data available on this pathology.

We report here the case of a 45-year-old woman with a large ovarian mucinous adenocarcinoma diagnosed during pregnancy, identified by ultrasound and magnetic resonance imaging.

The patient was treated by surgical resection followed by adjuvant chemotherapy with carboplatin and paclitaxel with a follow-up of 36 months, she is in complete remission.

## Highlights

• The association of ovarian malignancy with pregnancy is rare.
• The timing of delivery in patients with ovarian cancer again depends on stage and gestational age.
• The therapeutic approach should focus on a multidisciplinary team integrating gynecologic oncology, perinatology, and neonatology services.

## 1 Introduction

Ovarian cancer is the second most common gynecologic cancer associated with pregnancy [1,2]. Due to routine ultrasound examinations, the incidence of abdominal masses diagnosed during pregnancy is increasing and is estimated to be 2–10% of all pregnancies [1,3]. Functional cysts are the most common type of adnexal mass associated with pregnancy [4]. Only 3–6% of all pregnancy-associated ovarian cysts are malignant, and their diagnosis and management are complicated due to the lack of large randomized trials and cohort studies [2]. Malignant germ cell tumors are the most common ovarian tumors while epithelial to cancers are less frequently reported with an incidence of 1/12,000–1/50,000 of pregnancies [2,5]. There are no exact data on mortality from epithelial ovarian cancer during pregnancy; however, the prognosis is similar to that of non-pregnant patients [6]. Due to the rarity of cases and the limited data on this pathology; we hope to contribute to the study of this type of cancer, through a case of ovarian adenocarcinoma associated with a pregnancy of 19 weeks of gestation followed at the department of gynecology-obstetrics of the university hospital of Casablanca Morocco.

This work has been reported with respect to the SCARE 2020 criteria [7].

## 2 Case presentation

A 46-year-old woman with a history of thyroidectomy for thyroid goiter, on replacement therapy, third gesture, third pare, mother of two live children delivered by vaginal delivery, presented for an unattended pregnancy presumed at 5 months, who consulted for pelvic pain resistant to medical treatment with abdominal distension evolving for one month, without vomiting or transit disorders, without bleeding signs evolving in a context of altered general condition and weight loss not quantified.

The general examination on admission revealed a patient in poor general condition, asthenic, her blood pressure was 10/5 cmHg, her heart rate was 70 beats per minute.

The gynecological examination revealed an exaggerated uterine height for the term of the pregnancy, a long closed cervix of normal appearance with no bleeding and vaginal walls without anomalies.

**Fig. 1:**
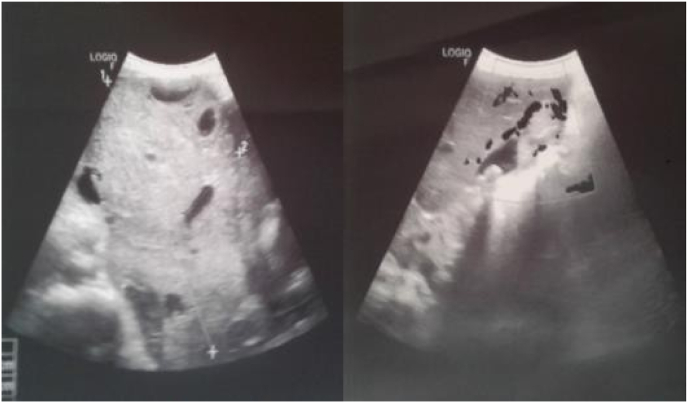
Abdominopelvic ultrasound scan showing a large abdominopelvic mass, reaching the uterus, oblong, echogenic, heterogeneous, vascularized on Doppler, with multiple poorly defined cystic formations, measuring 18x10 × 11 cm.

Abdominopelvic ultrasound showed a large abdominopelvic mass suspected of malignancy, reaching the uterus, oblong and echogenic, heterogeneous, vascularized on Doppler, with multiple poorly limited cystic formations, measuring 17x10 × 11 cm, with intrauterine presence of an evolving monofetal pregnancy estimated at 19 weeks of amenorrhea according to the biparietal diameter (Fig. 1).

**Fig. 2:**
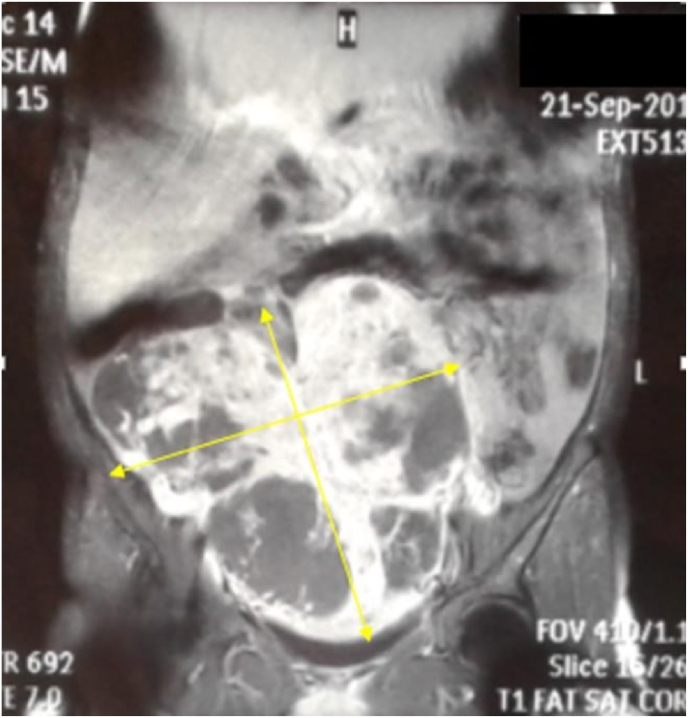
Pelvic MRI in coronal slices and T1 sequences showing a large solid cystic abdominal mass in T1 heterosignal, heterogeneously enhanced after gadolinium injection, measuring 220x100 × 175mm. This mass is poly-lobed and comes into contact with the uterus, the rectum and the bladder, with preservation of a separation line.

The CA 125 biomarker assay was elevated to 694 IU/ml. Pelvic MRI showed a large solid cystic abdominal mass with heterosignal T1, T2, heterogeneously enhanced after gadolinium injection, measuring 220x100 × 175mm. This mass is poly-lobed, well bounded, reaches the uterus and backwards to the rectum without obvious invasion. It pushes the bladder down and forward with preservation of a separation line. It is associated with a small peritoneal effusion with an intrauterine pregnancy (Fig. 2).

The joint decision of the multidisciplinary consultation staff and the couple was to carry out an exploratory laparotomy and radical surgery associated with medical interruption of pregnancy in case of malignant origin of the tumor.

**Fig. 3:**
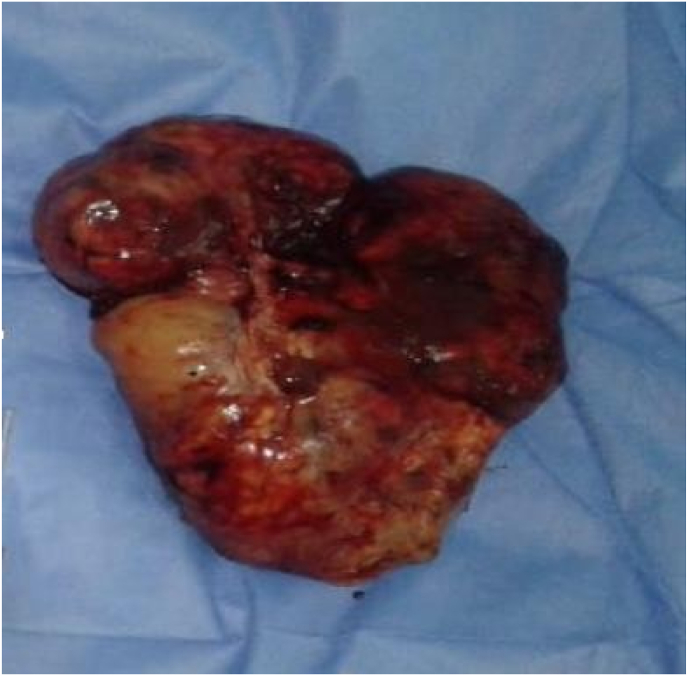
Macroscopic appearance of the surgical specimen of the left ovarian tumor.

The patient underwent an exploratory laparotomy with, at the time of surgical exploration, the presence of a large amount of ascites removed, a right ovarian mass 28 cm in diameter, friable, bleeding on contact with surface nodules adherent to the intestinal tract and the posterior surface of the uterus (Fig. 3).

A right adnexectomy was performed after a laborious adhesiolysis, the histological result after extemporaneous examination confirmed the diagnosis of moderately differentiated and invasive adenocarcinoma of the ovary, the hysterotomy section allowed the extraction of a female fetal death in utero weighing 550 g, the surgical procedure was completed by a total hysterectomy without adnexal preservation with omentectomy and peritoneal biopsy. The postoperative course was simple and the patient was discharged after 5 days.

**Fig. 4:**
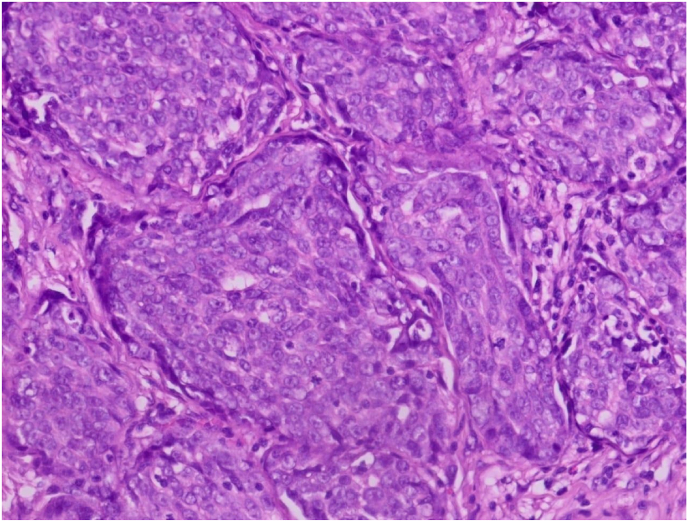
Adenocarcinomatous proliferation of the ovary at high magnification.

The definitive anatomopathological result of the surgical specimen confirmed ovarian mucinous adenocarcinoma of medium differentiation, the results of the epiploic and peritoneal biopsies were without signs of malignancy (Fig. 4).

The patient then received adjuvant chemotherapy with six courses of paclitaxel carboplatin. She has been followed up for three years in oncology consultations with follow-up assessments of CA 125 and abdomino-pelvic scans showing no signs of recurrence.

## 3 Discussion

Ovarian cancer is the second most common gynecologic cancer complicating pregnancy after cervical cancer [8,9].

The incidence of discovery of adnexal masses has increased with the routine use of ultrasound during pregnancy, of which approximately 3–6% are malignant [10].

The most common cases are ovarian malignancies diagnosed during pregnancy are germ cell, sex cord or borderline tumors and, less commonly, invasive epithelial cancers [11,12].

Prompt diagnosis of ovarian cancer during pregnancy is crucial for good therapeutic results, regardless of pregnancy status.

Unfortunately, the diagnosis of cancer during pregnancy is often delayed due to the difficulty in differentiating certain symptoms from those of the pregnant state; notably nausea, vomiting, breast changes, abdominal pain, anemia and fatigue [8]. These ovarian cancers are more frequently reported in primigravida, and the majority are diagnosed at an early stage by routine ultrasound routine ultrasound examinations [13,14].

Because of its high sensitivity and specificity in characterizing the morphology of abdominal masses, ultrasound examination is the optimal diagnostic tool during pregnancy, and it can also differentiate benign from malignant masses [12,15]. The malignant nature of ovarian tumors is indicated by several sonographic features such as size, solid component or complex appearance, papillary structure, internal septations, irregular margins, hyper-vascularization [16,17].

Ultrasound examinations are not able to differentiate benign tumors from tumors with low malignant potential; therefore, other imaging examinations are required [15]. MRI examinations can be safely performed in the second and third trimesters; in addition, they can also reveal possible extraovarian extension [2,17].

Biological markers such as CA125 are not contributory in pregnancy due to their physiological increase [13].

Therapeutic management of malignant tumors in pregnant women is challenging because of conflicts between optimizing maternal treatment and fetal well-being [13].

Both laparoscopic surgery and open laparotomy are acceptable surgical approaches. Care must be taken to avoid ovarian rupture. Staging surgery with preservation of the uterus and contralateral ovary is advised when possible.

In stage 1 or 2 disease, uterine preservation with peritoneal biopsies should be proposed as primary surgical treatment.

In patients with peritoneal extension of the disease, standard surgical management includes complete debulking surgery. This operation will necessarily terminate the pregnancy because a hysterectomy is required as in our patient's case [15].

If stage 3 cancer is diagnosed in the first or second trimester, it should be offered as a standard procedure. If continued pregnancy is chosen, debulking surgery, if necessary, should be performed after delivery [16].

Standard chemotherapy for epithelial ovarian tumors (a platinum-based agent with a taxane) can be used with minimal fetal risks in the second and third trimesters [17].

## 4 Conclusion

The timing of delivery in patients with ovarian cancer again depends on stage and gestational age.

The diagnosis of ovarian cancer during pregnancy is a devastating event accompanied by several dilemmas.

The therapeutic approach should focus on a multidisciplinary team integrating gynecologic oncology, perinatology, and neonatology services.

The results in our patient consolidate those in the literature in terms of the efficacy of The results in our patient consolidate those of the literature in terms of efficacy of the first radical surgery associated with the bi-chemotherapy based on platinum salt and taxanes.

## Ethical approval

I declare on my honor that the ethical approval has been exempted by my establishment.

## Sources of funding

None.

## Author contribution

Bouab Maryem: Corresponding author writing the paper.

## Consent

Written informed consent for publication of their clinical details and/or clinical images was obtained from the patient.

## Registration of research studies

None.

## Guarantor

DR BOUAB MARYEM.

## Provenance and peer review

Not commissioned, externally peer-reviewed.

## Declaration of competing interest

The authors declare having no conflicts of interest for this article.
